# Spiritual well-being and quality of life in maintenance hemodialysis patients: a latent profile analysis and serial mediation model

**DOI:** 10.3389/fpsyg.2026.1668699

**Published:** 2026-01-30

**Authors:** BiXia Yuan, QingHua Lai, Jing Wu, RongRong Wang, YongMei Lu, Meng Wu, Yun Chen, JieQian Wu

**Affiliations:** 1 Shenzhen Hospital (Futian) of Guangzhou University of Chinese Medicine, Shenzhen, China; 2 School of Nursing, Guangzhou University of Chinese Medicine, Guangzhou, China; 3 The Second Affiliated Hospital of Guangzhou University of Chinese Medicine, Guangzhou, China

**Keywords:** latent profile analysis, maintenance hemodialysis, psychosocial mediation, quality of life, spiritual well-being

## Abstract

**Background:**

From a positive psychology perspective, this study aimed to identify the latent profiles of spiritual well-being and analyze the serial mediation mechanism of family care and spiritual coping in the relationship between spiritual well-being and health-related quality of life (HRQoL). The findings are intended to inform strategies for improving the spiritual well-being of maintenance hemodialysis (MHD) patients.

**Methods:**

A cross-sectional design was employed with 220 MHD patients recruited from two tertiary hospitals in Guangdong, China (August 2023–January 2024). Assessments were conducted using the Functional Assessment of Chronic Illness Therapy–Spiritual Well-Being Scale (FACIT-SP-12), Family Care Index, Spiritual Coping Questionnaire (SCQ), and Short Form-12 Health Survey (SF-12). Latent profile analysis (LPA) was employed to identify heterogeneous subgroups based on spiritual well-being. A chain mediation model was then used to examine the mediating effects of family care and spiritual coping.

**Results:**

HRQoL scores averaged 56.50 ± 22.29. Significant correlations emerged: spiritual well-being (*r* = 0.557, *p* < 0.001), family care (*r* = 0.426, *p* < 0.001), and positive spiritual coping (*r* = 0.565, *p* < 0.001) positively predicted HRQoL, whereas negative coping correlated inversely (*r* = −0.343, *p* < 0.001). LPA identified four distinct profiles: Low spiritual well-being (36.8%), Moderate spiritual well-being (20.0%), Peaceful Mindset with Low Spiritual Belief (13.6%), and High spiritual well-being (29.5%). The mediation analysis revealed that family care and spiritual coping partially mediated the relationship between spiritual well-being and HRQoL (effect sizes: 0.074–0.175, 95% CI excluding 0). The chain mediation pathway “spiritual well-being → family care → spiritual coping → HRQoL” was statistically significant, with total indirect effects of 0.268 and 0.114 (95% CIs excluding 0).

**Conclusion:**

Spiritual well-being indirectly influences the quality of life in MHD patients through the serial mediation of family care and spiritual coping. Clinicians should recognize the heterogeneity in spiritual well-being and integrate routine spiritual screening into nursing assessments to identify patients with low spiritual well-being. It is recommended to develop family-based education and support programs, along with interventions that combine family care and spiritual coping strategies, so as to improve patients’ long-term health outcomes.

## Introduction

Maintenance hemodialysis (MHD) is currently the primary treatment modality for end-stage renal disease (ESRD), accounting for 89.5% of treated ESRD cases in China (Zhang and Cao, 2024). While advancements in medical care have improved the 5-year survival rate of MHD patients to 20–70% (Zhou et al., 2023), long-term dialysis imposes significant burdens, including physical discomfort, complications, financial strain, and psychological distress, which severely compromise patients’ quality of life. A recent analysis of health-related quality of life (HRQoL) in Chinese MHD patients observed lower HRQoL levels compared to population norms (Mai et al., 2023). Notably, impaired HRQoL has been identified as a significant predictor of adverse clinical outcomes in this population, as demonstrated by Mantzoukas et al. (2021).

Health-related quality of life refers to an individual’s perception of their position in life, influenced by cultural context, values, goals, expectations, and concerns, reflecting the impact of disease on health and daily living (Wang et al., 2025). Impaired HRQoL is highly prevalent among dialysis patients, with MHD patients experiencing multidimensional challenges that compromise physical, psychological, and social functioning (Zhi et al., 2025). Therefore, during MHD treatment, it is of significant importance to explore ways to help patients improve their HRQoL and facilitate their maximum reintegration into society.

Spiritual well-being refers to a state in which an individual affirms the meaning of life, recognizes the value of oneself, others, and the environment, connects harmoniously with them, and possesses the inner strength to transcend limitations (Xiao et al., 2025). As an internal resource that supports adaptation, spirituality plays a crucial role in helping individuals cope with illness (Gholami et al., 2021). Patel et al. (2002) reported a relationship between spirituality and health outcomes, indicating that spirituality positively influences patients’ health perceptions and their ability to cope with serious illnesses. Compared to the general population, patients undergoing MHD exhibit significantly impaired quality of life in terms of physical health, social functioning, and psychological well-being (Joshi et al., 2017). Fradelos et al. (2022) further revealed that MHD patients generally exhibit lower levels of spiritual well-being, which significantly correlates with poorer quality of life. Despite growing evidence on factors influencing HRQoL in MHD patients, the mechanisms through which spiritual well-being influences HRQoL remain poorly understood.

Family care, a critical component of social support systems (Shi et al., 2023), provides both practical assistance and emotional sustenance to MHD patients, thereby promoting health improvement and quality of life. However, limited studies have explored the interplay between spiritual well-being, family care, and HRQoL in this population. Existing literature suggests a positive correlation between spiritual well-being and family care (Yuan et al., 2024), and studies in cardiac populations indicate that family care significantly enhances quality of life (Zhang et al., 2021). These findings underscore the need to investigate tailored interventions integrating spiritual and family-centered support for MHD patients.

Spiritual well-being significantly influences the selection of spiritual coping strategies, which are cognitive-behavioral approaches through which patients utilize spiritual resources to manage illness-related stressors (Liu et al., 2023). Spirituality is an intrinsic resource of the individual that permeates all dimensions of life and is inherent in every person, regardless of religious affiliation. In general, spiritual coping encompasses two types of strategies: “positive” and “negative” coping (Saffari and Chen, 2019). The spiritual coping framework proposed by Gall et al. (2005) describes how individuals engage their spiritual resources to navigate the challenges of illness, thereby achieving a state of holistic well-being across physical, psychological, social, and spiritual. Within this framework, spirituality functions as a set of coping resources rooted in faith and values that individuals may employ when facing stress. These resources include spiritual appraisal, spiritual coping strategies, spiritual connection, and meaning-making.

**Figure 1:**
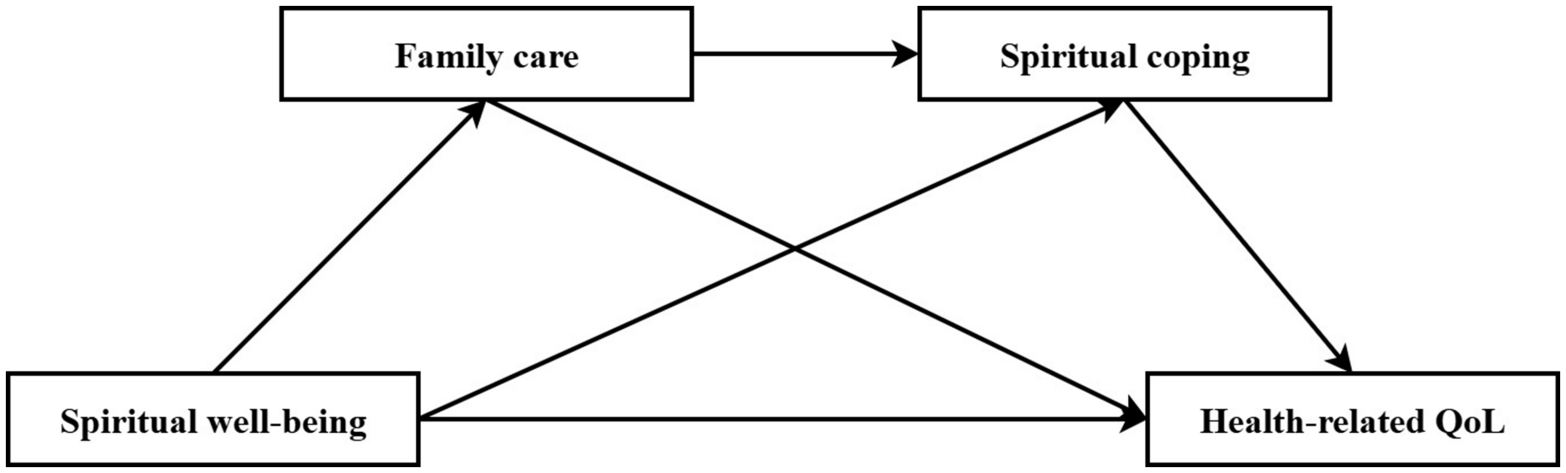
The conceptual model.

Guided by this framework, we position *spiritual connection* (operationalized as family care) and *spiritual coping* (positive and negative strategies) as mediators to explore the relationship between *spiritual appraisal* (spiritual well-being) and health outcomes (HRQoL). Additionally, latent profile analysis (LPA) is used to examine heterogeneity in spiritual well-being among MHD patients. This approach aims to inform tailored spiritual and quality-of-life interventions for clinical practice. We propose the following hypotheses (see Figure 1 for details):

*H1*: Spiritual well-being, family care, spiritual coping, and HRQoL are significantly correlated.

*H2*: Spiritual well-being among MHD patients is heterogeneous and can be categorized into distinct latent classes with different characteristics through LPA.

*H3*: Spiritual well-being directly predicts HRQoL.

*H4*: Family care mediates the relationship between spiritual well-being and HRQoL

*H5*: Family care and spiritual coping sequentially mediate the relationship between spiritual well-being and HRQoL (chain mediation).

## Methods

### Participants

A convenience sampling method was used to recruit patients from the hemodialysis centers of two tertiary hospitals in Guangdong Province, China, between August 2023 and January 2024. Inclusion criteria: (1) age ≥18 years; (2) patient met the diagnostic criteria of stage 5 chronic kidney disease following the clinical guidelines of the US Kidney Disease Prognosis Quality Initiative (Levey et al., 2002); (3) receiving regular hemodialysis 2–3 times weekly for ≥3 months with stable condition; (4) ability to communicate and complete questionnaires independently; (5) voluntary participation. Exclusion criteria: (1) history of mental illness or intellectual disability; (2) acute critical comorbidities.

The sample size was estimated using Kendall’s principle (Gorsuch, 1997). With a total of 33 variables in this study, a sample size of 5–10 times the number of variables was targeted, and a 10% attrition rate was factored in. This yielded a required sample size of 182–363 participants. Ultimately, 228 questionnaires were distributed, and 220 valid responses were collected, resulting in a valid response rate of 96.5%.

### Data collection procedure

This study was approved by the ethics committee of The Second Affiliated Hospital, Guangzhou University of Chinese Medicine (Grant number: YM2023-219). Two trained investigators administered the questionnaires using standardized instructions. Participants were informed of the study’s purpose, confidentiality, their right to withdraw at any time, and informed consent was obtained. Questionnaires were completed independently by patients before dialysis sessions to avoid interference with treatment. For participants unable to self-complete, investigators verbally administered items and recorded responses. The survey process lasted 15–20 min, and questionnaires were reviewed immediately for completeness, with missing items addressed promptly.

## Measures

### Demographic and clinical characteristics

Data included gender, age, religious affiliation, marital status, education level, employment status, household monthly income per capita, medical payment method, dialysis vintage, and number of chronic comorbidities.

### Functional assessment of chronic illness therapy-spiritual well-being scale (FACIT-SP-12)

The Chinese version of the FACIT-SP-12 was used to assess spiritual well-being (Bredle et al., 2011). This 12-item scale comprises three dimensions (*peace*, *meaning*, and *faith*) rated on a 5-point Likert scale (0 = “not at all” to 4 = “very much”). Items 4 and 8 were reverse-scored. Total scores range from 0 to 48, categorized as *low* (0–23), *moderate* (24–35), or *high* (≥36) spiritual well-being. Cronbach’s *α* coefficient for this scale was 0.950 in the current study.

### Family care index

The Family Care Index, originally developed by Smilkstein et al. and adapted into Chinese by Lv and Gu (1995), comprises five dimensions: family adaptation, partnership, growth, affection, and resolve. Each item is rated on a 3-point Likert scale (0 = “rarely,” 2 = “often”), with total scores ranging from 0 to 10. Higher scores indicate better family functioning. In this study, the scale demonstrated excellent internal consistency (Cronbach’s *α* = 0.873).

### Chinese version of the spiritual coping questionnaire (SCQ)

The SCQ, developed by Charzyńska (2015) based on a multidimensional conceptualization of spirituality, was translated into Chinese to assess both religious and non-religious spiritual coping strategies. It includes two subscales: positive spiritual coping (personal, social, environmental, and transcendental dimensions) and negative spiritual coping (personal, social, and transcendental dimensions), totaling 26 items across seven dimensions. Items are rated on a 5-point Likert scale (1 = “very inaccurate” to 5 = “very accurate”). Subscale scores are calculated as the mean of item responses, with higher scores indicating greater reliance on specific coping strategies. Internal consistency was high for both subscales (positive coping: *α* = 0.884; negative coping: *α* = 0.908).

### Short Form-12 health survey (SF-12)

The SF-12, derived from the SF-36 by Ware et al. (1996), assesses HRQoL across eight domains grouped into two summary scores: *Physical Component Summary* (PCS; includes general health [GH], physical functioning [PF], role limitations due to physical health [RP], and bodily pain [BP]) and *Mental Component Summary* (MCS; includes role limitations due to emotional problems [RE], mental health [MH], vitality [VT], and social functioning [SF]). The total score is standardized to a 0–100 scale, with higher scores reflecting better quality of life. The scale showed strong reliability in this study (Cronbach’s *α* = 0.913).

### Statistical analysis

All statistical analyses in this study were performed using IBM SPSS 25.0, the SPSS PROCESS macro v4.1, and Mplus 8.3. First, general demographic and clinical variables were described using means ± standard deviations (mean ± SD) or frequencies (percentages). Pearson correlation analysis was employed to examine the relationships among spiritual well-being, family care, spiritual coping, and quality of life. Next, LPA was used to identify potential spiritual well-being subgroups. Latent profile models were established by progressively testing 1–6 class solutions. The model fit was evaluated using the following indices, as proposed by Williams and Kibowski (Williams and Kibowski, 2016): the Akaike Information Criterion (AIC), Bayesian Information Criterion (BIC), sample-size-adjusted BIC (aBIC), entropy, the Lo–Mendell–Rubin likelihood ratio test (LMR), and the bootstrap likelihood ratio test (BLRT). Lower values of AIC, BIC, and aBIC indicate better model fit. Entropy represents classification accuracy, ranging from 0 to 1; an entropy value ≥ 0.8 indicates a classification accuracy > 90%. A significant *p*-value (< 0.05) for the LMR or BLRT suggests good model fit.

Finally, Model 4 of the SPSS PROCESS macro was used to assess the mediating roles of family care and spiritual coping between spiritual well-being latent classes (categorical variable) and quality of life. Model 6 of the PROCESS macro was applied to test the serial mediation effects of family care and spiritual coping on the relationship between spiritual well-being (continuous variable) and quality of life. The bias-corrected percentile bootstrap method (5,000 resamples, 95% CI) was used to evaluate the total, direct, and indirect effects of the models. An indirect effect was considered statistically significant if its 95% confidence interval did not include zero (Berkovits et al., 2000).

## Results

### Descriptive statistics

Among the 220 MHD patients (130 males, 90 females), the majority were aged ≤60 years (109, 49.5%), had no religious affiliation (180, 81.8%), were married (172, 78.2%), had a middle school education (75, 34.0%), and were unemployed (169, 76.8%). Most patients had medical insurance (214, 97.3%), a household monthly income >5,000 CNY (86, 39.1%), a dialysis duration of 12–60 months (97, 44.1%), and 3–4 chronic comorbidities (114, 51.8%). The mean HRQoL score was 56.50 ± 22.29. Significant differences in HRQoL were observed across age (*p* = 0.004), religious status (*p* = 0.003), education level (*p* = 0.013), employment status (*p* = 0.005), and number of comorbidities (*p* = 0.003) (see Table 1).

**Table 1:** Demographic and clinical characteristics of MHD patients (*N* = 220).

Variable	*n* (%)	HRQoL (Mean ± SD)	*t/F*	*p*
Gender			1.02	0.309
Male	130 (59.1)	57.77 ± 21.63		
Female	90 (40.9)	54.66 ± 23.21		
Age (years)			5.81	0.004
45	42 (19.1)	62.01 ± 18.73		
45 ~ 60	79 (35.9)	60.47 ± 20.79		
>60	99 (45.0)	50.99 ± 23.73		
Religious affiliation			3.02	0.003
Yes	40 (18.2)	65.96 ± 19.94		
No	180 (81.8)	54.40 ± 22.29		
Marital status			2.04	0.133
Unmarried	19 (8.6)	63.51 ± 19.26		
Married	172 (78.2)	56.75 ± 22.18		
Divorced/widowed	29 (13.2)	50.44 ± 23.88		
Education level			3.23	0.013
Primary school or below	55 (25.0)	50.21 ± 22.18		
Middle school	75(34.1)	54.62 ± 20.10		
High school/technical	50 (22.7)	59.38 ± 23.77		
College	19 (8.6)	61.88 ± 21.87		
Bachelor’s or above	21 (9.5)	67.95 ± 22.25		
Employment status			2.84	0.005
Employed	51	64.13 ± 19.40		
Unemployed	169	54.20 ± 22.64		
Household income (CNY)			1.30	0.277
<1,000	26 (11.8)	50.18 ± 16.96		
1,001 ~ 3,000	39 (17.7)	59.26 ± 18.16		
3,001 ~ 5,000	69 (31.4)	54.73 ± 26.36		
>5,000	86 (39.1)	58.58 ± 21.67		
Medical payment			1.13	0.34
Medical insurance	178 (80.9)	57.33 ± 22.03		
New rural cooperative	27 (12.3)	51.59 ± 20.64		
Public funding	9 (4.1)	61.68 ± 30.07		
Self-payment	6 (2.7)	46.03 ± 24.26		
Dialysis vintage			1.14	0.332
<1 year	68 (30.9)	54.34 ± 22.80		
1–5 years	97 (44.1)	55.59 ± 21.46		
5–10 years	37 (16.8)	62.39 ± 20.98		
>10 years	18 (8.2)	57.47 ± 26.81		
Chronic comorbidities			5.99	0.003
<3	90 (40.9)	62.60 ± 21.48		
3 ~ 4	114 (51.8)	52.42 ± 21.95		
>4	16 (7.3)	51.22 ± 22.49		

### Common method Bias test

As self-administered questionnaires may introduce common method bias, Harman’s single-factor test (Tang and Wen, 2020) was conducted to assess potential bias. Exploratory factor analysis (EFA) of all study variables revealed 12 factors with eigenvalues >1. The first factor accounted for 32.65% of the variance (below the 40% threshold), indicating no significant common method bias or multicollinearity.

### Multivariate linear regression analysis of factors influencing HRQoL in MHD patients

This study explored the determinants of HRQoL in MHD patients through hierarchical multivariate linear regression analysis (see Table 2). After sequentially incorporating demographic characteristics, spiritual well-being, family care, and spiritual coping strategies into the models, the adjusted *R*^2^ increased significantly from 0.11 (Model 1) to 0.42 (Model 4). The results identified spiritual well-being (*β* = 0.51, *p* < 0.01) as the strongest predictor of HRQoL, with its effects partially mediated through family care (*β* = 0.30, *p* < 0.01) and positive spiritual coping strategies (*β* = 0.28, *p* < 0.01). In contrast, age (*β* = −0.10, p < 0.01) and number of chronic comorbidities (*β* = −0.15, *p* < 0.05) were negatively associated with HRQoL, while negative spiritual coping did not reach statistical significance (*β* = −0.11, *p* > 0.05). These findings suggest that interventions aimed at enhancing spiritual well-being, strengthening family support systems, and fostering adaptive coping strategies may improve long-term quality of life in MHD patients.

**Table 2:** Multivariate linear regression analysis of factors influencing HRQoL in MHD patients (*n* = 220).

Model	Variable	*β*	*SE*	Standardized *β*	*F*	*R* ^2^
Model 1	Age	−2.8	2.15	−0.10	6.52**	0.11
	Religious affiliation	10.18**	3.69	0.18		
	Education level	2.79*	1.31	0.15		
	Employment status	2.44	3.90	0.05		
	Number of comorbidities	−5.45*	2.42	−0.15		
Model 2	Age	−0.17	1.94	−0.01	16.55**	0.30
	Religious affiliation	2.05	3.45	0.04		
	Education level	0.61	1.20	0.03		
	Employment status	0.50	3.47	0.01		
	Number of comorbidities	−2.68	2.18	−0.07		
	Spiritual well-being	0.93**	0.12	0.51		
Model 3	Age	−2.08	1.88	−0.07	19.25**	0.37
	Religious affiliation	4.07	3.30	0.07		
	Education level	0.12	1.14	0.01		
	Employment status	0.85	3.29	0.02		
	Number of comorbidities	−3.70	2.08	−0.10		
	Spiritual well-being	0.68**	0.13	0.37		
	Family care	2.49**	0.50	0.30		
Model 4	Age	−2.151	1.83	−0.07	18.29**	0.42
	Religious affiliation	2.547	3.23	0.04		
	Education level	0.343	1.11	0.02		
	Employment status	1.354	3.17	0.03		
	Number of comorbidities	−3.744	2.03	−0.10		
	Spiritual well-being	0.33**	0.15	0.18		
	Family care	1.72**	0.52	0.20		
	Positive spiritual coping	0.47**	0.13	0.28		
	Negative spiritual coping	−0.37	0.20	−0.11		

*p < 0.05; **p < 0.01.

### Latent profile analysis of spiritual well-being in MHD patients

**Figure 2:**
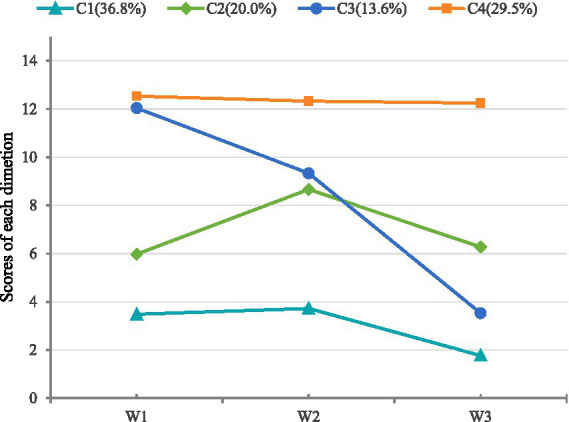
Score probabilities of four latent profiles of spiritual well-being in MHD patients.

As shown in Table 3, for the 1–6 profile models, the entropy values were all above 0.80, and as the number of classes increased, the AIC, BIC, and aBIC gradually decreased, indicating better model fit with more classes. The entropy and LMR test results showed that the 4-class model outperformed the 3-class model (*p* < 0.05). When the number of classes was increased to 5 or 6, the LMR test results were no longer significant (*p* > 0.05), suggesting that adding more classes did not significantly improve model fit. Although the 2-class model showed higher entropy and LMR values than the 4-class model, the 4-class model offered better theoretical interpretability and more clearly captured the heterogeneity in spiritual well-being among MHD patients. Therefore, after considering both model fit indices and clinical interpretability, the 4-class model was selected as the optimal latent profile model for this study. The four latent classes C1 ~ C4 are named as: Low Spiritual Well-Being (36.8%), Moderate Spiritual Well-Being (20.0%), Peaceful Mindset with Low Spiritual Belief (13.6%), and High Spiritual Well-Being (29.5%) (see Figure 2 for details).

**Table 3:** Fit indices of latent profile models for spiritual well-being in MHD patients (*n* = 220).

Model	1	2	3	4	5	6
LL	−1936.717	−1785.774	−1750.936	−1724.862	−1709.438	−1701.974
AIC	3885.433	3591.548	3529.871	3485.724	3462.876	3455.948
BIC	3905.795	3625.484	3577.382	3546.81	3537.536	3544.182
aBIC	3886.781	3593.794	3533.016	3489.768	3467.818	3461.788
Entropy	—	0.884	0.816	0.849	0.827	0.844
LMR(P)	—	<0.0001	0.0428	0.0057	0.1869	0.3938
BLRT(P)	—	<0.001	<0.001	<0.001	<0.001	0.0500
Group sizes (%)						
C1	220 (100%)	115 (52.3%)	84 (38.2%)	81 (36.8%)	46 (20.9%)	62 (28.2%)
C2	—	105 (47.7%)	73 (33.2%)	44 (20.0%)	70 (31.8%)	52 (23.6%)
C3	—	—	63 (28.6%)	30 (13.6%)	36 (16.4%)	24 (10.9%)
C4	—	—	—	65 (29.5%)	30 (13.6%)	26 (11.8%)
C5	—	—	—	—	38 (17.3%)	21 (9.5%)
C6	—	—	—	—	—	35 (15.9%)

### Validation of the mediation models involving family care and spiritual coping

The results revealed significant correlations among quality of life, spiritual well-being, family care, positive spiritual coping, and negative spiritual coping (*p* < 0.01 for all), detailed in Table 4. Using the Low Spiritual Well-Being group (as a reference category in the latent profile analysis of spiritual health), detailed in Table 5, the mediation effects of family care was estimated as 0.122, 0.217, and 0.227. The corresponding 95% bootstrap confidence intervals were (0.258, 0.873), (0.170, 0.885), and (0.732, 1.301), none of which contained zero, demonstrating a significant mediating effect. Similarly, the mediation effects of spiritual coping were 0.186, 0.0895, and 0.248. The 95% bootstrap confidence intervals were (0.026, 0.330), (0.009, 0.186), and (0.035, 0.439), respectively. As none of these intervals included zero, spiritual coping also demonstrated a significant mediating effect. Detailed pathways are illustrated in Table 5.

**Table 4:** Correlation analysis among spiritual well-being, family care, spiritual coping, and health-related quality of life in MHD patients (*n* = 220).

	Correlation							
	Mean	SD	1	2	3	4	5	6
1. HRQoL	56.5	22.29	1					
2. Spiritual well-being	21.77	12.2	0.557**	1				
3. Family care	7.63	2.65	0.426**	0.360**	1			
4. Spiritual coping	72.51	13.14	0.403**	0.552**	0.209**	1		
5. Positive spiritual coping	55.8	13.43	0.565**	0.685**	0.408	0.874**	1	
6. Negative spiritual coping	16.72	6.68	−0.343**	−0.292**	−0.408**	0.210**	−0.292**	1

**p < 0.01.

**Table 5:** Mediation effects of spiritual well-being latent classes (categorical variable) on health-related quality of life (HRQoL).

Mediation pathway	Effect (95%CI) 1VS.2	Effect (95%CI) 1VS.3	Effect (95%CI) 1VS.4
Model 1			
Direct effect: spiritual well-being → HRQoL	0.566 (0.258, 0.873)	0.528 (0.170, 0.885)	1.016 (0.732, 1.301)
Indirect effect: spiritual well-being → family care → HRQoL	0.122 (0.019, 0.250)	0.217 (0.087, 0.369)	0.227 (0.111, 0.371)
Model 2			
Direct effect: spiritual well-being → HRQoL	0.501 (0.158, 0.845)	0.655 (0.290, 1.021)	0.995 (0.662, 1.329)
Indirect effect: spiritual well-being → Spiritual coping → HRQoL	0.186 (0.026, 0.330)	0.0895 (0.009, 0.186)	0.248 (0.035,0.439)

### Chain mediation effect analysis

**Figure 3:**
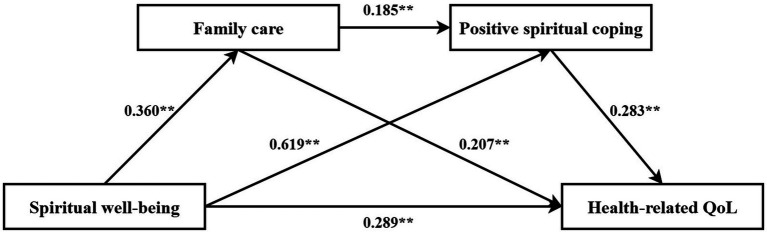
Chain mediation effect of family care and positive spiritual coping on health-related quality of life (HRQoL).

**Figure 4:**
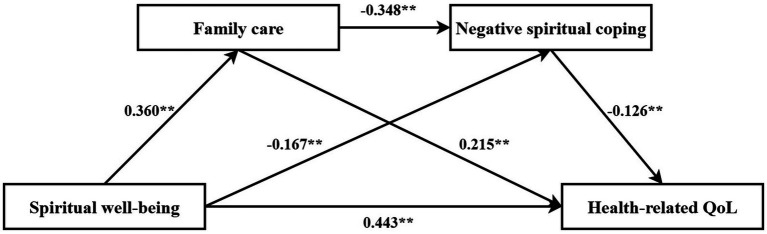
Chain mediation effect of family care and negative spiritual coping on health-related quality of life (HRQoL).

Two chain mediation models were constructed with spiritual well-being (continuous variable) as the independent variable, family care and positive/negative spiritual coping as mediators, and quality of life as the dependent variable, as illustrated in Figures 3, 4. The bias-corrected percentile bootstrap method (5,000 resamples) was used to validate the chain mediation effects of family care and spiritual coping on spiritual well-being and quality of life, with 95% confidence intervals (CIs). The results, detailed in Table 6, demonstrated that the total, direct, and indirect effects of spiritual well-being on quality of life were all statistically significant (95% CIs excluded zero). Specifically, spiritual well-being indirectly influenced quality of life through family care (effect sizes: 0.074 and 0.077; 95% CIs: [0.032, 0.126] and [0.031, 0.132]) and through spiritual coping (effect sizes: 0.175 and 0.021; 95% CIs: [0.074, 0.264] and [0.001, 0.051]). Furthermore, the chain mediation pathway via both family care and spiritual coping significantly impacted quality of life (effect sizes: 0.019 and 0.016; 95% CIs: [0.004, 0.040] and [0.001, 0.051]). See Table 6 for details.

**Table 6:** Chain mediation effects of family care and spiritual coping on spiritual well-being and health-related quality of life (HRQoL).

Path	Effect	*SE*	LLCI	ULCI
Model 1				
Total effect	0.557	0.056	0.446	0.668
Direct effect	0.289	0.072	0.146	0.432
Ind1: spiritual well-being → family care → HRQoL	0.074	0.024	0.032	0.126
Ind2: spiritual well-being → positive spiritual coping → HRQoL	0.175	0.048	0.074	0.264
Ind3: spiritual well-being → family care → positive spiritual coping → HRQoL	0.019	0.009	0.004	0.040
Total indirect effect	0.268	0.053	0.155	0.366
Model 2				
Total effect	0.557	0.056	0.446	0.668
Direct effect	0.443	0.058	0.328	0.558
Ind1: spiritual well-being → family care → HRQoL	0.077	0.026	0.031	0.132
Ind2: spiritual well-being → negative spiritual coping → HRQoL	0.021	0.013	0.001	0.051
Ind3: spiritual well-being → family care → negative spiritual coping → HRQoL	0.016	0.008	0.002	0.033
Total indirect effect	0.114	0.029	0.063	0.174

## Discussion

Spiritual well-being, as a critical component of holistic health, has been recognized as a universal trait through which individuals seek meaning in life and has been consistently linked to improved quality of life (Nair and Kumar, 2025). In this study, we identified heterogeneity in spiritual well-being among MHD patients, with spiritual well-being directly influencing health-related quality of life. Family care and spiritual coping strategies partially mediated this relationship. Furthermore, the established chain mediation models demonstrated that family care and spiritual coping sequentially mediate the pathway between spiritual well-being and HRQoL.

### Correlations among spiritual well-being, family care, spiritual coping, and HRQoL in MHD patients

The findings revealed a positive correlation between spiritual well-being and HRQoL in MHD patients, suggesting that inner peace, a sense of purpose, and spiritual beliefs contribute to better health outcomes (Batra et al., 2024). Family care, a key element of social support systems, not only directly enhanced HRQoL but also partially mediated the relationship between spiritual well-being and HRQoL, underscoring its role in fostering adaptive spiritual health and coping strategies (Zhou et al., 2023). Higher levels of family care provided patients with greater resources to mitigate disease-related distress, promote positive health behaviors, and reframe their perspectives on illness, thereby improving quality of life (Iida et al., 2025). Spiritual coping influenced HRQoL through dual pathways of psychological adaptation and behavioral modification (Bonilla Sierra et al., 2025). Specifically, positive spiritual coping strengthened adaptive behaviors to enhance HRQoL, whereas negative spiritual coping exacerbated feelings of helplessness, further deteriorating HRQoL.

### Latent class analysis of spiritual well-being in MHD patients

Previous studies have predominantly assessed patients’ spiritual well-being based on total scores, often overlooking population heterogeneity (Babamohamadi et al., 2020). This study employed latent profile analysis to categorize the spiritual well-being of MHD patients into four distinct groups: Low Spiritual Well-Being Group (36.8%), Moderate Spiritual Well-Being Group (20.0%), Peaceful Mindset-Low Spiritual Belief Group (13.6%), and High Spiritual Well-Being Group (29.5%). Notably, the combined proportion of patients in the low and moderate spiritual well-being groups exceeds 50%. Furthermore, the analysis revealed that patients in the Peaceful Mindset-Low Spiritual Belief Group exhibited high scores on the *peace* dimension but low scores on the *faith* dimension. This indicates that some patients maintain a state of inner calmness yet lack spiritual conviction or the ability to draw support from religious beliefs, a phenomenon that may be related to the relatively low prevalence of religious affiliation in China.

However, spirituality is culturally specific, and religion represents only one facet of spiritual resources (Xu et al., 2023). Traditional Chinese philosophies, such as Confucianism and Taoism, can serve as intrinsic spiritual resources, providing patients with psychological support and resilience (Wang, 2024). Healthcare professionals should guide patients to correctly understand spirituality and provide tailored spiritual care based on their specific spiritual well-being profile and personal cultural background. This care can be delivered through various modalities, such as music therapy (Zhang et al., 2025), advance care planning (Sellars et al., 2018), and life review therapy (Huang et al., 2020). Additionally, approaches that transcend the physical form, including meditation, spiritual guidance, and faith-based rituals, can be utilized (Yangöz et al., 2025). The goal of these interventions is to help patients enhance their sense of meaning in life, improve their capacity to utilize spiritual resources, and ultimately achieve a state of holistic well-being encompassing physical, psychological, social, and spiritual.

### Mediating roles of family care and spiritual coping

The findings indicate that family care mediates the relationship between spiritual well-being and HRQoL in MHD patients, suggesting that spiritual well-being can indirectly influence the quality of life of dialysis patients through the mediating role of family care. Family functioning—defined as the capacity of family members to provide practical, financial, and emotional support—enhances both individual and family resilience to life stressors and plays a vital role in health outcomes (Gallardo-Peralta et al., 2022). MHD imposes significant disruptions on patients’ work, diet, and lifestyle. Family care acts as a protective factor, offering much-needed assistance and emotional support to help patients adapt to new routines, alleviate stress, and foster proactive disease management (Mohammadi et al., 2024). However, in practice, family care may exhibit a “double-edged sword” effect. Excessive protective behaviors can undermine patients’ self-efficacy, while the long-term economic burdens and emotional exhaustion associated with caregiving may gradually erode the quality and sustainability of support (You et al., 2025; Chen et al., 2025). Therefore, future research should distinguish between facilitative dimensions of family care (e.g., emotional support, respect for autonomy) and potentially constraining dimensions (e.g., over-involvement, relational burden). Longitudinal studies are needed to track the dynamic evolution of these dimensions across different stages of illness and examine their impact on patient adaptation outcomes.

The results also show that spiritual well-being influences the quality of life of MHD patients through the mediation of positive spiritual coping. That is, MHD patients with higher levels of spiritual well-being tend to employ more positive spiritual coping strategies, which in turn are associated with better quality of life. Additionally, negative spiritual coping mediates the relationship between spiritual well-being and quality of life, with higher spiritual well-being negatively predicting the use of negative coping strategies, thereby indirectly affecting quality of life. As an important internal resource for managing life stress, spiritual well-being helps patients cope with pressures and encourages the adoption of positive spiritual coping strategies. Individuals with lower spiritual well-being have fewer spiritual coping resources at their disposal, perceive greater stress, and are more prone to developing negative coping patterns (Xia, 2025). Furthermore, this study adopted the Chinese-adapted version of the Spiritual Coping Scale as the measurement tool. Although the scale underwent a certain degree of linguistic and cultural adaptation during its introduction, its items were originally developed within a Western religious and cultural context. Therefore, the scale may still have limitations in fully capturing the diverse indigenous spiritual concepts in China (Li, 2022) (such as *Tian Dao* [the Way of Heaven], *yuanfen* [predestined affinity], and *xiuxing* [self-cultivation]). During administration, we observed that some participants naturally integrated their own cultural understanding and traditional beliefs when interpreting certain items. This vividly illustrates how spiritual coping embodies unique meanings and vitality across different cultural contexts, and it also offers insights for future efforts to develop spiritual coping research frameworks that incorporate multicultural perspectives.

### Chain mediation effects of family care and spiritual coping between spiritual well-being and quality of life in MHD patients

The findings of this study further demonstrate that spiritual well-being also influences quality of life through a chain mediation pathway involving family care and spiritual coping. The spiritual coping process model posits that under stress, individuals engage in a sequential process involving spiritual appraisal, spiritual connection, and spiritual coping, which ultimately manifests in psychophysiological outcomes. This theory explains the chain mediation effect observed here: MHD patients facing illness experience stress; their spiritual well-being represents their cognitive spiritual appraisal; family care serves as an external spiritual connection; spiritual coping reflects their tendency to choose specific spiritual coping strategies; and the patient’s quality of life constitutes the resulting outcome of this stress response. Research indicates that individuals with low spiritual well-being struggle to mobilize internal positive spiritual coping resources when facing stressors (Hu et al., 2023). They are prone to biased cognition and processing of emotional stimuli, easily becoming entrenched in negative affective states, which can lead to poor mental health outcomes. In contrast, family care is a crucial protective factor for psychological well-being. Enhancing the level of family care can reduce the occurrence of patients’ negative emotions, thereby improving the quality of life of MHD patients.

However, current clinical practice in spiritual care predominantly focuses on a patient-centered model, often failing to fully acknowledge the critical role of the family in spiritual support (Jiang and Fang, 2022). Spiritual assessment is typically limited to the patient’s individual spiritual state, and interventions primarily target the patient alone, lacking systematic attention to the family’s capacity for spiritual support. Therefore, it is necessary to shift spiritual care from an individual-oriented approach to a relationship-oriented one. Specifically, at the assessment level, spiritual well-being measurement tools applicable to the patient-family should be developed to identify specific barriers families face in providing spiritual support. At the intervention level, family intervention programs should be designed—for example, guiding families to establish daily communication patterns that incorporate spiritual dimensions. Simultaneously, healthcare professionals should receive professional training to enhance their competence as facilitators and coordinators of family spiritual communication. By working with family members to guide patients toward positive spiritual coping strategies, they can help mitigate the negative impacts of the disease and ultimately improve patients’ quality of life.

### Limitations

This study has several limitations. First, the sample was limited to Chinese MHD patients, which may affect the generalizability of the findings to other populations. Second, the cross-sectional design precludes causal inferences. Future studies should adopt longitudinal designs to establish temporal relationships. Third, the sample was drawn from a relatively concentrated source and was limited in size, which may cause selection bias. Fourth, the use of self-report questionnaires may be subject to subjective bias, such as social desirability or recall bias. Fifth, the included variables did not account for clinical indicators or potential psychological confounders. Future research would benefit from longitudinal designs, multi-center sampling, and the inclusion of objective clinical measures alongside multidimensional assessments to address these limitations.

## Data Availability

The original contributions presented in the study are included in the article/supplementary material, further inquiries can be directed to the corresponding author.
